# Analysis of FRP-Strengthened Reinforced Concrete Beams Using Electromechanical Impedance Technique and Digital Image Correlation System †

**DOI:** 10.3390/s23218933

**Published:** 2023-11-02

**Authors:** Ricardo Perera, María Consuelo Huerta, Marta Baena, Cristina Barris

**Affiliations:** 1 Department of Mechanical Engineering, Technical University of Madrid, 28006 Madrid, Spain; mariaconsuelo.huerta@upm.es; 2 Analysis and Advanced Materials for Structural Design (AMADE), Polytechnic School, University of Girona, 17003 Girona, Spain; marta.baena@udg.edu (M.B.); cristina.barris@udg.edu (C.B.)

**Keywords:** FRP strengthening, electromechanical impedance (EMI), digital image correlation (DIC), structural health monitoring (SHM)

## Abstract

Fiber-reinforced polymer (FRP) strengthening systems have been considered an effective technique to retrofit concrete structures, and their use nowadays is more and more extensive. Externally bonded reinforcement (EBR) and near-surface mounted (NSM) technologies are the two most widely recognized and applied FRP strengthening methods for enhancing structural performance worldwide. However, one of the main disadvantages of both approaches is a possible brittle failure mode provided by a sudden debonding of the FRP. Therefore, methodologies able to monitor the long-term efficiency of this kind of strengthening constitute a challenge to be overcome. In this work, two reinforced concrete (RC) specimens strengthened with FRP and subjected to increasing load tests were monitored. One specimen was strengthened using the EBR method, while for the other, the NSM technique was used. The multiple cracks emanating in both specimens in the static tests, as possible origins of a future debonding failure, were monitored using a piezoelectric (PZT)-transducer-based electromechanical impedance (EMI) technique and a digital image correlation (DIC) system. Clustering approaches based on impedance measurements of the healthy and damaged states of the specimens allowed us to suspect the occurrence of cracks and their growth. The strain profiles captured in the images of the DIC system allowed us to depict surface hair-line cracks and their propagation. The combined implementation of the two techniques to look for correlations during incremental bending tests was addressed in this study as a means of improving the prediction of early cracks and potentially anticipating the complete failure of the strengthened specimens.

## 1. Introduction

Within the field of strengthening and rehabilitating reinforced concrete (RC) civil structures, the use of fiber-reinforced polymer (FRP) composite materials is more and more extensive [1,2,3]. FRPs provide numerous advantages, such as high tensile strength, light weight, the ability to customize the FRPs’ properties, and good long-term durability. Two effective strengthening techniques using FRPs, externally bonded reinforcement (EBR) [4,5] and near-surface mounted (NSM) [6,7,8,9] reinforcement, have appeared in the last decades as rising methodologies to enhance the strength and stiffness of existing RC structures. In the EBR approach, an FRP laminate is glued onto the tensile face of the RC with epoxy resin, while in NSM strengthening, an FRP bar or strip is inserted into longitudinal grooves previously cut in the concrete cover and bonded therein with either epoxy adhesives or cement mortars. The performance of this kind of FRP strengthening may be altered by the mechanical and environmental conditions the structure must support. Regardless of the strengthening technique, the failure of the strengthened member is often caused by FRP reinforcement debonding that can take place in different zones of the member, either at the end of the external reinforcement or at the location of bending/shear cracks. This kind of failure occurs in a sudden and brittle way, which, if not detected in its earliest stages, can have catastrophic consequences. Because of this, to guarantee the confidence of owners in the future use of these materials, effective methods for condition assessment should be implemented [10,11,12].

In the recent past, works based on the electromechanical impedance (EMI) technique with the use of PZT transducers have been carried out to monitor crack initiation and growth in FRP-strengthened concrete structures [13,14,15,16,17,18]. By permanently bonding low-cost transducers to a structure, this can be interrogated in a remote and wireless framework when desired since PZTs can act as both actuators and sensors, yielding an active-sensing technique that is able to detect internal damage at a low cost from the measurement of electrical impedances. In addition, as PZT transducers work in a high-frequency range, the EMI technique would be very useful for assessing the health of areas near to each sensor.

Although the sensitivity of the EMI technique is in the order of that of other local techniques, such as ultrasonic testing, it can be considered a global dynamic technique. For the identification of minor cracks, its combination with other types of measurements might contribute to improving the accuracy and efficiency of the procedure, especially in terms of defect localization. For instance, static strain measurements are very sensitive to responses in the vicinity of local defects [19,20]. In this sense, a non-time-consuming methodology that is able to provide full-field measurements of strains might complement the EMI technique to expedite the prediction of early cracks as the possible origins of a future debonding failure.

The digital image correlation (DIC) technique is a non-contact optical numerical measuring system that can provide full-field measurements of displacements and strains by comparing sequential images of the inspected region captured with a digital camera via the loading procedure [21,22,23]. In comparison with other traditional methods of measurement, such as LVDTs and strain gauges, which require direct contact with the specimen surface and provide only pointwise measurements, DIC allows quickly obtaining full-field measurements of displacements and strains in the tested structures.

Digital image correlation has been widely used not only in structural health monitoring applications [24,25] but also in other applications such as the characterization of materials’ mechanical behavior [26,27]. Its applications in concrete structures are also numerous, mainly including crack detection and growth [23,28] as well as the identification of fracture properties and failure mechanisms [29,30].

However, the DIC approach only captures surface images of concrete structures. This means internal cracking and phenomena cannot be identified even though they might become crucial for the failure of the analyzed specimen, as would be the case in the intermediate debonding of FRP-strengthened RC beams. This disadvantage might be reduced with the EMI methodology since it allows us to identify inner anomalies. Therefore, as commented in the third paragraph of this section, the joint application of DIC and EMI technologies could be very advantageous in some applications.

In this work, the combination of the EMI technique, based on the processing of impedance signatures, and the DIC technique, based on the processing of images, is addressed as a means of improving the methodology of identification of minor and early cracks in FRP-strengthened structures. The response of a structure is characterized by simultaneously measuring the impedance change in the PZT sensor and the strains induced in the specimen during the loading procedure. As the cracks are expected to alter the strain field in their vicinity as well as the electromechanical impedance in the PZT sensors, it is interesting to explore the advantages provided by their combined effects.

## 2. Principle of EMI Technique

The EMI technique is a local technique based on smart piezoelectric materials such as PZT (lead zirconate titanate oxide) patches. PZTs are very sensitive, light, and non-expensive materials, which, when bonded onto the surface of an infrastructure, allow its monitoring.

Piezoelectric materials have the capability to convert electrical energy into mechanical energy and vice versa. These materials generate an electric charge upon applying stress (direct effect) and exhibit mechanical deformation upon applying an electric field (converse effect):

(1) $$\left[D\right]=\left[d\right]\left[T\right]+\left[\varepsilon^{T}\right]\left[E\right]$$

(2) $$\left[S\right]=\left[s^{E}\right]\left[T\right]+\left[d\right]\left[E\right]$$

where [D] is the electric displacement vector, [S] the second-order strain tensor, [E] the applied external electric field vector, and [T] the stress tensor. Accordingly, [ε^T^] is the second-order dielectric permittivity tensor under constant stress, [d] the piezoelectric coefficient tensor, and [s^E^] the fourth-order elastic compliance tensor under a constant electric field.

Equation (1) shows the direct piezoelectric effect, while Equation (2) implies the converse effect. This is the reason why PZTs act as sensors as well as actuators.

In the EMI technique, the same PZT is used as the actuator and sensor in such a way that the structure is excited with the sensor using the converse piezoelectric effect, and the mechanical response is captured with the same PZT using the direct piezoelectric effect. Liang et al. [31] were the first to propose a one-dimensional model of a PZT sensor bonded to a structural system. In this model, the PZT sensor is simplified as a bar connected to the structure, which is simulated as a mass with stiffness and damping. Under a high-frequency excitation, the system experiences axial simple harmonic vibration. By using the direct and converse piezoelectric effects of PZT sensors, the variations in structural mechanical impedance due to damage are represented with EM admittance measurements, Y(ω), whose expression is denoted as follows:

(3) $$Y\left(\omega \right)=j\omega \frac{wl}{h}\left({\bar{\varepsilon }}_{33}^{T}-\frac{Z_{s}\left(\omega \right)}{Z_{s}\left(\omega \right)+Z_{a}\left(\omega \right)}d_{3x}^{2}{\hat{Y}}_{xx}^{E}\right)$$

where Z_s_ and Z_a_ are the mechanical impedance of the host structure and the PZT sensor, respectively; w, l, and h are the width, length, and height of the PZT sensor, respectively; ${\bar{\varepsilon }}_{33}^{T}=\varepsilon_{33}^{T}\left(1-\delta j\right)$ and ${\hat{Y}}_{xx}^{E}$ are the electrical permittivity under zero electric field and the complex Young’s modulus of the PZT under zero stress, respectively; δ denotes the dielectric loss factor of the PZT; and d_31_ is a coupling piezoelectric coefficient.

In the EMI technique, the RMSD value as a scalar damage metric has been widely accepted for assessing damage by estimating the variation between two impedance signatures. It can be calculated using the following equation:

(4) $$RMSD\left(\%\right)=\sqrt{\frac{\sum_{i=1}^{N}{\left[Re\left(Z_{1}\left(\omega_{i}\right)\right)-Re\left(Z_{0}\left(\omega_{i}\right)\right)\right]}^{2}}{\sum_{i=1}^{N}{Re\left(Z_{0}\left(\omega_{i}\right)\right)}^{2}}}\cdot 100$$

where Re(Z_0_(ω_i_)) is the real part of the impedance or conductance measured with the PZT sensor before the structure is damaged, and Re(Z_1_(ω_i_)) is the corresponding conductance after damage at the ith measurement point. The RMSD value often increases as the severity of damage increases. The conductance is often more sensitive to structural damage than the imaginary part of the impedance.

### Hierarchical Clustering Method

The use of a single type of metric, such as RMSD (Equation (4)), for EMI-based damage detection has some limitations that affect the identification of the damage location and severity. Non-model-based clustering methods are able to correlate the location and type of PZT transducer with its performance as well as with the level of damage of the structure to be analyzed. Basically, clustering is an unsupervised learning algorithm that is able to organize objects into groups according to their similarity or some recognized pattern.

Measurements captured with one sensor at different stages of the inspected structure should show similar behavior, except if some anomaly has occurred. Potential anomalies might be identified at similar stages for the same sensor.

In this work, a hierarchical clustering algorithm was applied. By using this hierarchical clustering approach, each one of the different measurements captured with one PZT sensor is initially treated as a single cluster. Subsequently, using an iterative procedure, nearby observations are merged into a new bigger cluster. Then, successively, new nearby points are added to the nearest group, and so on. A bottom-up or agglomerative strategy is used. The different levels of grouping can be visualized using a dendrogram, which is a sort of connectivity plot giving a big picture of the level of similarity between adjacent clusters.

## 3. Experimental Set-Up

**Figure 1:**
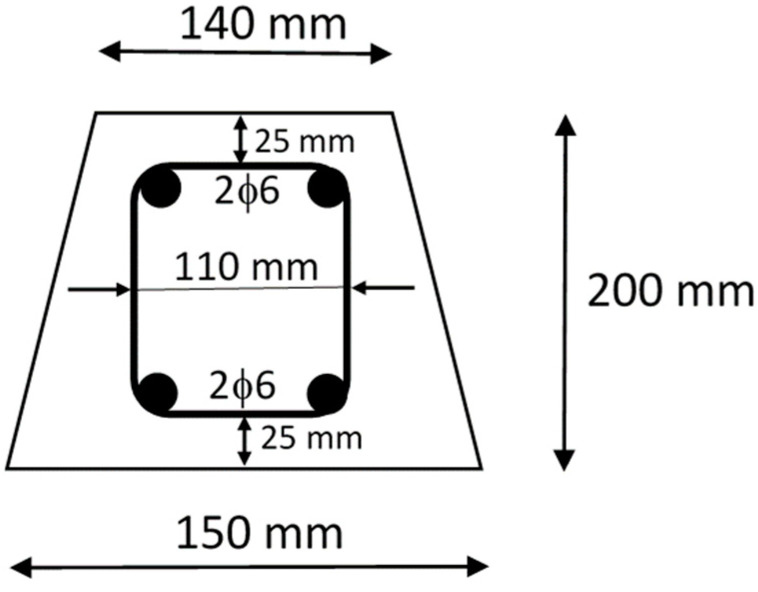
Specimen cross-section.

The tested specimens were two reinforced beams with trapezoidal cross-sections (Figure 1) and a length of 1.9 m. The top and bottom reinforcements consisted of two steel bars with a diameter of 6 mm. Steel stirrups with a diameter of 6 mm were used as shear reinforcement. They were placed at a distance of 100 mm along the length of the beam with a cover of 25 mm.

For both specimens, the material properties of the concrete and the reinforcement steel were as follows: (a) concrete: compressive strength (fc) = 35 MPa, and tensile strength (fct) = 3 MPa; (b) steel: yield strength (fy) = 500 MPa, and elastic modulus (Es) = 210 GPa. One beam, Specimen 1, was externally strengthened by bonding a 1430 mm length × 50 mm width × 1.2 mm thickness CFRP plate (Sika CarboDur S512, Sika, Alcobendas, Spain) onto the lower tensile face of the concrete, while the other, Specimen 2, was strengthened with a 1430 mm length × 8 mm diameter CFRP bar (MasterBrace BAR 165/2500, Sika, Cornellá, Spain) inserted on its cover following the NSM method. The properties of the CFRP material were as follows: (a) Specimen 1: tensile strength (ffu) = 3100 MPa, and elastic modulus (Ef) = 170; (b) Specimen 2: ffu = 2500 MPa, and Ef = 165 GPa.

In the test program that was performed, the strengthened beams were subjected to a series of four-point increasing symmetric static load tests with the purpose of gradually introducing deterioration into the specimens. A steel beam with a span of 300 mm was placed on the test beam to distribute the symmetrical load. For each loading step, the beam was subjected to three cycles of loading and unloading, reaching the charge defined for each step at the end of every loading. The purpose of this method was to stabilize the cracks that appeared on the beam after each loading step. The programmed loads reached in each loading step for both specimens are shown in Table 1 and Table 2, respectively.

The performance of both specimens was monitored with PZT patches as well as a DIC system whose set-up will be shown in the next sections.

### 3.1. PZTs’ Set-Up

**Figure 2:**
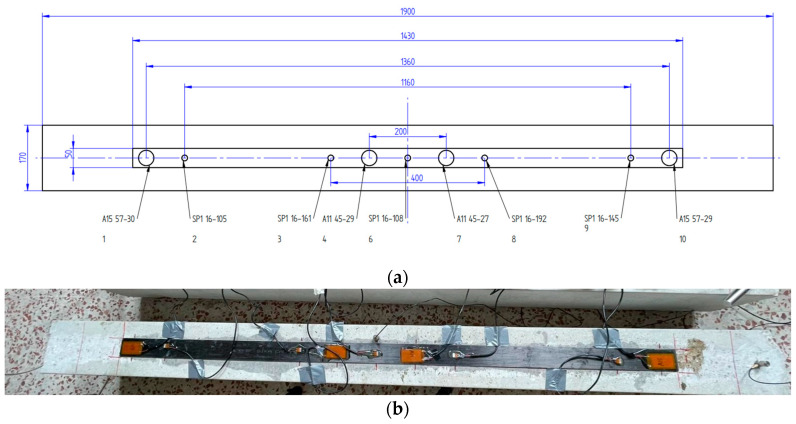
PZTs’ set-up for Specimen 1 (dimensions in mm): (**a**) Detailed scheme; (**b**) Photo.

Figure 2 shows the distribution of the PZTs on the tensile face of Specimen 1. All PZT patches were bonded directly to the FRP strip. P-876.A11 and P-876-SP1 types were used, except for close to the ends of FRP, where P-876.A15 (61 mm × 35 mm × 0.8 mm) sensors were glued [32].

**Figure 3:**
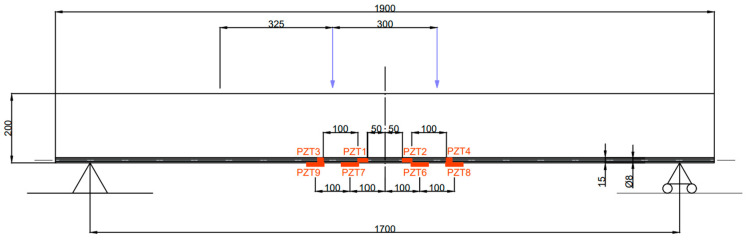
PZTs’ set-up for Specimen 2 (dimensions in mm).

Figure 3 shows the PZTs’ set-up for Specimen 2. According to this figure, four 61 mm × 35 mm × 0.4 mm PZT patches, PZT6–PZT9, were glued to the concrete tensile surface and correspond to sensors of the P-876.A11 DuraAct Patch type. The PZT1–PZT4 sensors were bonded directly to the FRP sheet. PZT1 and PZT2 correspond to P-878-A1 (27 mm × 9.4 mm × 0.6 mm), while PZT3 and PZT4 are of the P-876-SP1 type (16 mm × 13 mm × 0.5 mm).

With the purpose of measuring the change in the impedance across the different stages of the beam, the terminals of the PZTs were attached to an impedance analyzer (Agilent HP 4192A, Agilent Technologies, CA, USA) to acquire the EMI signatures. The EMI signatures were acquired in the range of 10–100 kHz, with a step interval of 12.5 kHz.

### 3.2. DIC System Configuration

Digital image correlation is an optical measurement technique that is widely used in experimental mechanics to compute the deformation of a body [33,34]. The DIC technique was used in this work to consistently acquire the full field of displacements and strains of the two analyzed specimens with the purpose of better understanding their progressive cracking and the effect on the final failure at the concrete–FRP interface. A 2D configuration was used for all specimens. DIC technology is based on using template matching to track objects. An initial area of randomly distributed spots on the surface of the specimen or speckle image is defined as a template in the first image of a sequence of video frames. By using a template-matching technique, the template can be located in successive images. The displacements of the template are measured based on the pixel coordinates and converted into the physical displacements of the object using a scaling factor. Different approaches can be used for the image correlation process [34,35,36].

The DIC system was installed in front of one of the side surfaces of the specimen for the detection of any crack propagation during the progressive loading stages.

**Figure 4:**
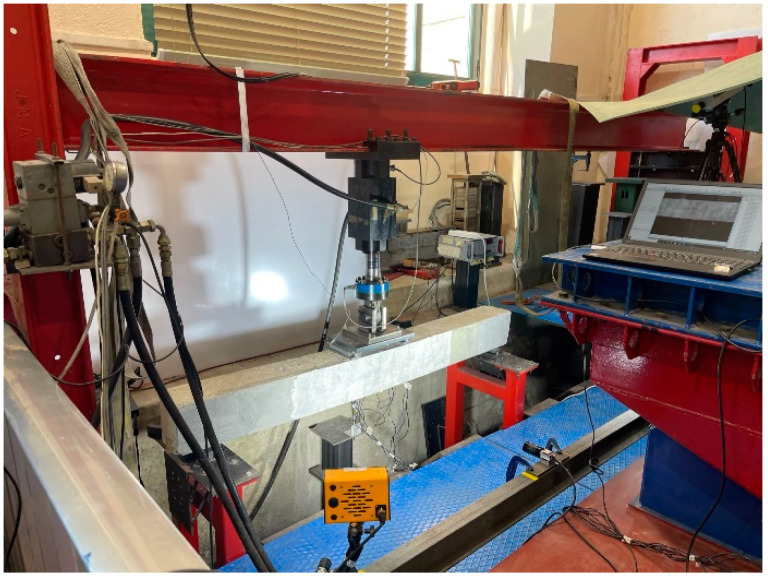
DIC system set-up.

The setup of the DIC system is shown in Figure 4, which includes a CCD industrial camera (BASLER ac4112, Ahrensburg, Germany, 4096 × 3000 pixels, 15 mm lenses), two combined LED illumination panels (GSVitec LT, IN, USA), and a computer to control image acquisition. The LED panels improved the contrast intensity of the speckle pattern, providing sufficient lighting for the DIC measurements independent of ambient conditions.

The camera, mounted on a tripod, was aligned perpendicular to the front surface at a working distance of approximately 750 mm, and a field-of-view (FoV) of 600 mm × 200 mm was achieved, which allowed the cracks on the front beam to be viewed.

As the surface contrast textures were weak, prior to the beginning of the tests, a speckle pattern on the front face of the beam was created to establish the image correlation. For this, a layer of white paint was first sprayed on the surface of the beam as a background. Once it dried, black matte color paint was sprayed on the background to generate the speckle pattern.

The capture of the speckle pattern images was performed with the Vic-Snap software 1.1 (SC, USA) installed on the control computer. A timed capture method was adopted, and the capture speed was 20 frames/min with the digital camera.

VIC-2D 6.0 (SC, USA) software was used for the DIC calculations and computation of Lagrangian strain maps from the digital images captured only on the side surface of the specimen being photographed. Full-field displacements could be retrieved by comparing the digital images using subset-based local algorithms. The displacements in the thickness direction were not considered as they were assumed to be relatively small. The first image without loading was set as a reference. A subset of 45 × 45 pixels with a step size of 7 pixels was chosen to analyze the strain of the specimen.

## 4. Results and Discussion

### 4.1. Specimen 1

**Figure 5:**
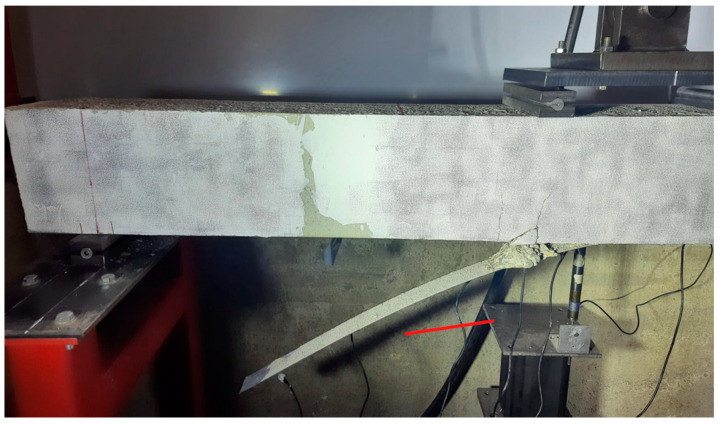
FRP debonding—Specimen 1.

For Specimen 1, during the loading procedure, the first vertical crack was noted during Test 3 (Table 1), on the bottom side in the constant-moment zone. Then, scattered flexural tension cracks were formed first in this moment zone and, subsequently, between the loading points and the supports. This multiple crack formation was the catalyst for the strengthened beam’s failure. The failure mode of this specimen occurred via FRP debonding induced by an intermediate crack formed close to the left loading point and propagation toward the left end of the strip (Figure 5).

To simplify this study, from the 17 tests shown in Table 1, we will focus only on Tests 1, 2, 3, 6, 7, 8, 9, 10, 12, 16, and 17. Then, to refer to them in the following figures, a test code (1 to 11) is assigned in the third column of Table 1.

The response of the specimen was characterized by simultaneously measuring the electromechanical impedance change in the PZT sensors as well as the principal longitudinal strain induced in the beam during bending testing.

**Figure 6:**
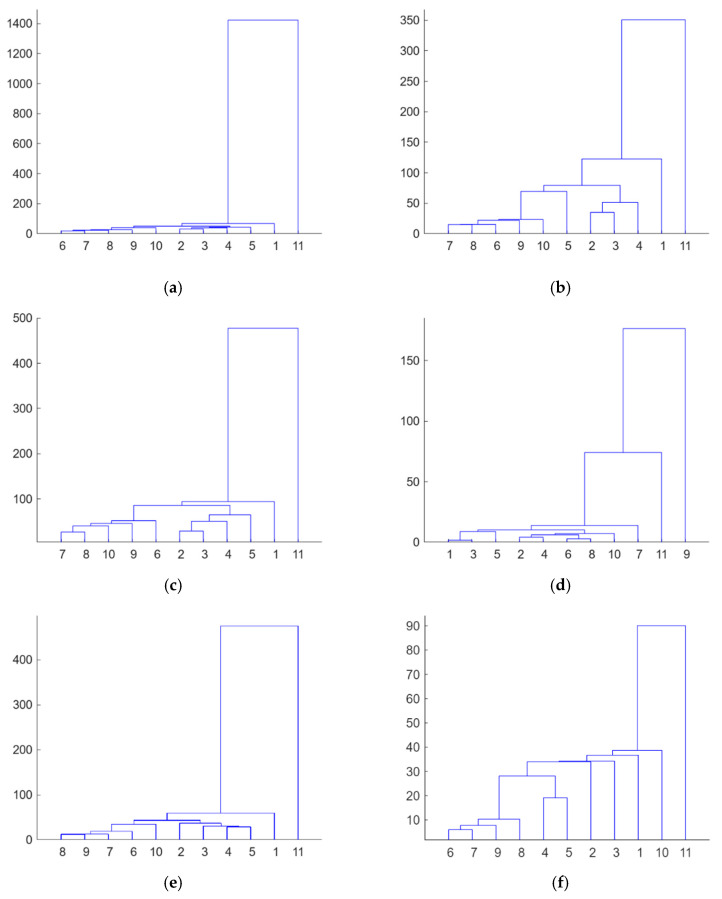

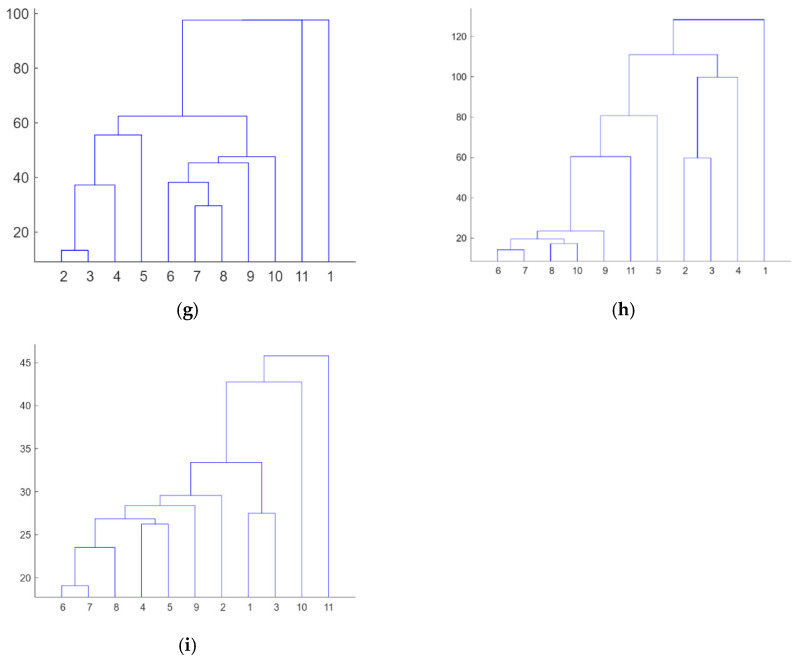
Hierarchical trees—Specimen 1: (**a**) PZT1; (**b**) PZT2; (**c**) PZT3; (**d**) PZT4; (**e**) PZT6; (**f**) PZT7; (**g**) PZT8; (**h**) PZT9; and (**i**) PZT10.

**Figure 7:**
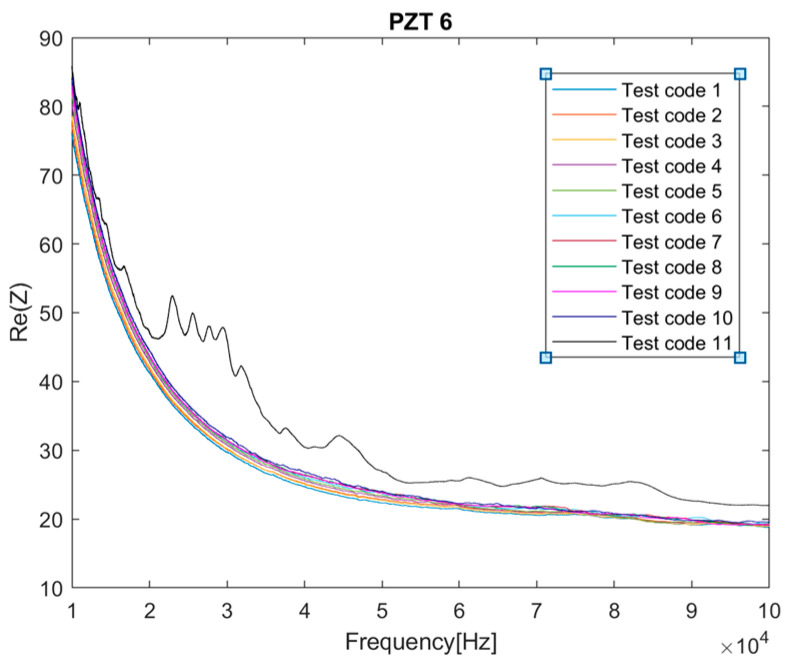
Evolution of the impedance signal for PZT6 sensor.

Firstly, from the dataset of electromechanical impedance signatures captured with the PZTs through the different loading stages, an unsupervised hierarchical clustering analysis [13,17] was separately applied to each sensor to build the different clusters formed according to the similarity or dissimilarity between the different load or damage scenarios. Figure 6 shows the dendrograms obtained from this analysis. A dendrogram consists of many U-shaped lines that connect data points in a hierarchical tree. The lengths of the stems (the height of each U) of the dendrograms represent the variation among the different identified groups, i.e., the distance or dissimilarity between the two data points being connected. According to this, it is clear that Sensors 1 to 8, except Sensor 4, clearly discriminate the stage (the test with code 11) just before the failure of the beam. These sensors are those located in the intermediate region of the beam, where multiple cracks occurred, or close to the left end of the strip, where failure propagated from intermediate regions. Figure 7 shows the evolution of the real part of the impedance for Sensor 6 in the different loading stages. The last test clearly provides an impedance different from that of the other tests. This conclusion is not extensible to Sensors 9 and 10, the closest sensors to the right end of the FRP strip, since in this zone, the FRP-to-concrete bonded joint was not altered by FRP debonding. However, observing Figure 6d, although Test 11 is also discriminated, something noticeable should have also occurred for Sensor 4 during Test 9. It is difficult to predict the origin of this warning using only the EMI approach, but it might indicate the creation of a significant crack close to Sensor 4 or another anomaly.

**Figure 8:**
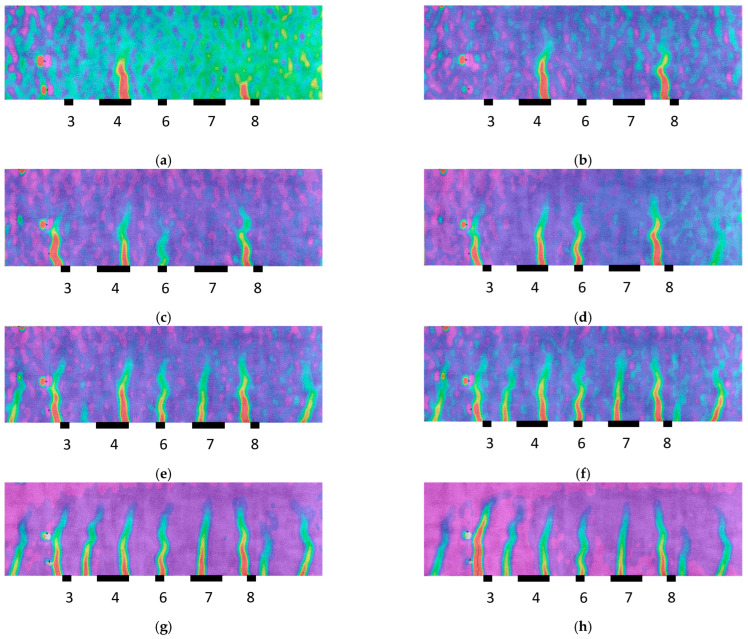
Strain map—Specimen 1: (**a**) test code 4; (**b**) test code 5; (**c**) test code 6; (**d**) test code 7; (**e**) test code 8; (**f**) test code 9; (**g**) test code 10; and (**h**) test code 11.

**Figure 9:**
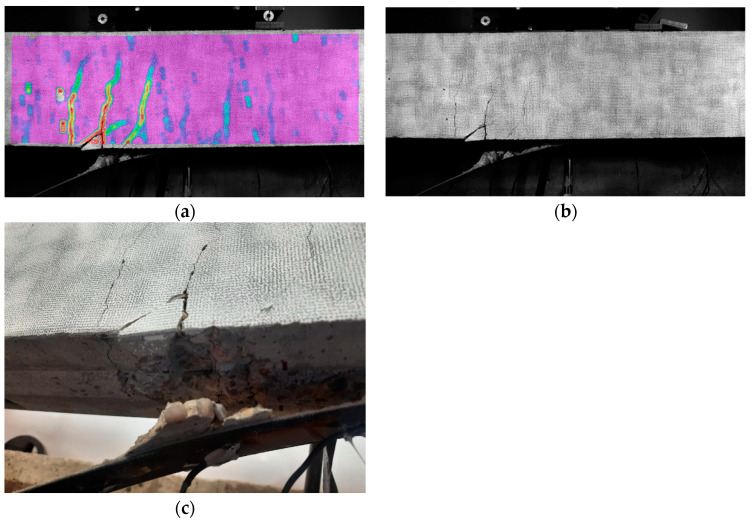
Failure mode—Specimen 1: (**a**) DIC image; (**b**) frontal photo; and (**c**) detail of the failure.

The longitudinal strain maps for the different loading steps in Table 1 or damage evolution stages were also extracted in the region of interest of the specimen using the DIC system and are shown in Figure 8. The images of the DIC correspond to the test code in Table 1 identified at the foot of each image, and the locations of the PZT sensors are also shown in the figures. The red color indicates maximum tensile strains. Cracks and, therefore, damage locations were identified on the contour maps, where significant increases in the strain, marked in red in the figures, were observed. The growth in the strain localization, visually non-observable but automatically detected with the DIC methodology, indicates the crack growth trajectories of the specimen. The DIC images show the initial cracks around Sensor 4 in the constant-moment zone (Figure 8a) and the subsequent propagation to areas closer to the other PZT sensors until the failure of the specimen (Figure 9), which occurred just after Test 11 in Figure 8h.

Now, to better understand the damage development until failure in this kind of FRP strengthening, cross-validation of the experimental results obtained with EMI and image correlation was carried out for the different levels of load. As damages are expected to change the strain field in their vicinity, and, consequently, alter the impedance, the integration of both monitoring methods might contribute to the implementation of a robust and successful method for the assessment of this type of structure.

Firstly, it is clear the strain profiles measured with DIC in the region of interest perfectly justify the distribution of the dendrograms for the different PZT sensors. The irruptions of cracks captured with DIC in the vicinity of the sensors are consistent with the PZT responses obtained in similar conditions, such as in Figure 6. In general terms, although not in a strict sense and with some exceptions, there is a slight division between the performance of the tests with codes 2 to 5 and tests with codes 6 to 10, which agrees with the crack distributions provided via DIC. Test 11 clearly shows a considerable change in impedance for all the sensors located in the region of interest, which is logical because it represents the previous instant to the failure of the specimen.

However, it is interesting to remark that for Sensor 4, a significant variation in impedance was also observed for the test with code 8, which might reveal the appearance of an important anomaly during this test. Upon inspecting the strain map associated with this test (Figure 8e), nothing anomalous was detected with respect to the previous test. However, in the following test (Figure 8f), an important new crack appeared close to Sensor 4, which might be responsible for this extra change in impedance. The fact that a 2D DIC methodology was used means that only surface cracks may be identified with this approach. However, this anomaly was already detected in the previous test (Figure 6d) with the fourth PZT sensor. The cause of this discrepancy might be due to this new damage originating on the lower face of the beam before propagating to the side face. Because of this, it was detected earlier with the PZT sensor than with the DIC methodology.

The sudden irruption of this newly deteriorated area might be the origin or warning of a possible future failure of the specimen. This is confirmed by Figure 9, where the failure mode of the beam is shown. Effectively, failure occurred via the sudden debonding of the FRP strip, but its origin was in the vicinity of this area. Additionally, Figure 9c shows that part of the concrete cover on the lower face was stripped off during this failure, which agrees with the reasoning of the previous paragraph.

In conclusion, combining EMI and DIC to investigate the damage in EBR FRP-strengthened beams was found to be fruitful in monitoring their global evolution and identifying the different stages of their failure mechanism.

### 4.2. Specimen 2

Specimen 2 corresponds to an RC beam strengthened with the NSM FRP methodology, i.e., an FRP strip was inserted into the concrete cover. Compared with externally bonded FRP reinforcement, NSM reinforcement is less prone to debonding from the concrete substrate, which is particularly attractive for the flexural strengthening of beams.

For this specimen, a similar study to that in Section 4.1 was carried out. However, although several cracks grew during the loading procedure, the failure of the specimen could not be reached because due to the limitations of the hydraulic jack, only a maximum load of 100 kN could be applied. This demonstrates the higher efficiency of the NSM FRP method compared with the EBR FRP method.

**Figure 10:**
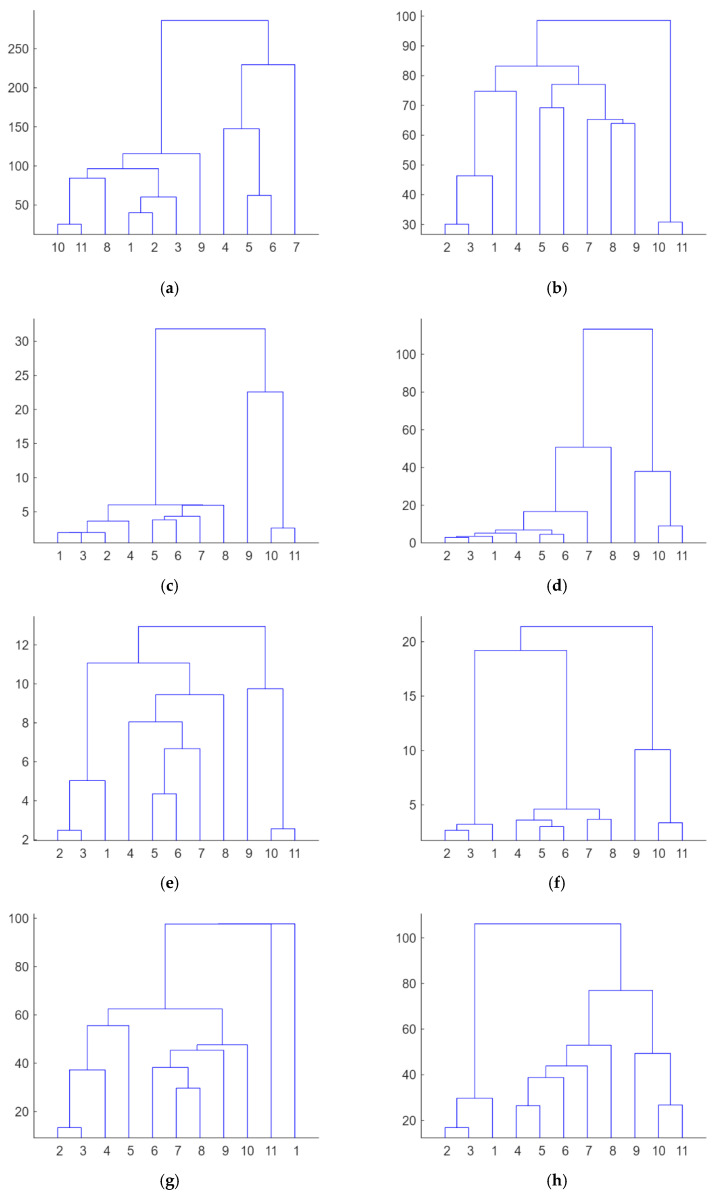
Hierarchical trees—Specimen 2: (**a**) PZT1; (**b**) PZT2; (**c**) PZT3; (**d**) PZT4; (**e**) PZT6; (**f**) PZT7; (**g**) PZT8; and (**h**) PZT9.

**Figure 11:**
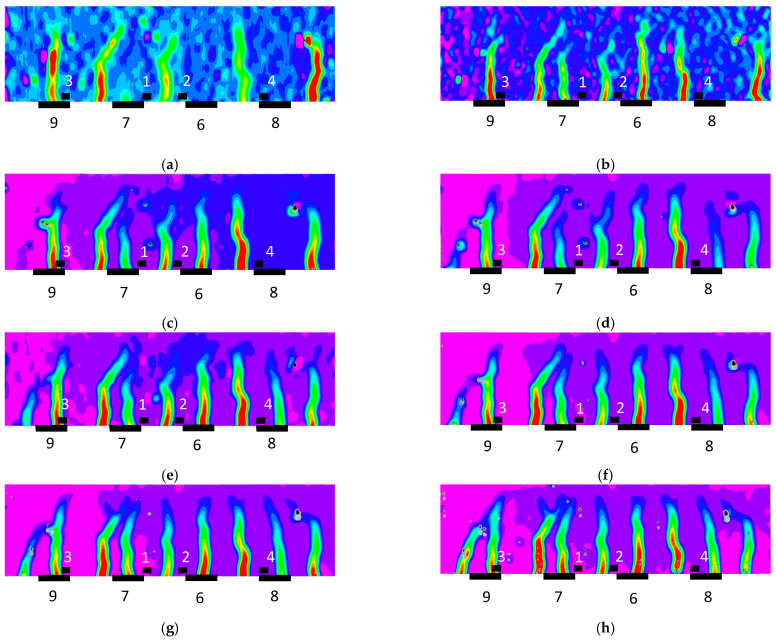
Strain map—Specimen 2: (**a**) Test 3; (**b**) Test 4; (**c**) Test 5; (**d**) Test 6; (**e**) Test 7; (**f**) Test 8; (**g**) Test 9; and (**h**) Test 10.

Figure 10 shows the dendrograms constructed for each sensor, and Figure 11 shows the distribution of the longitudinal strain in the DIC system. To extract the images in Figure 11, a higher level of opacity than in Figure 8 was used. A strain map was not obtained for Test 11.

The crack distribution of Test 3 (Figure 11a) differs clearly from those of the next tests. This is captured in the dendrograms, especially those corresponding to the external sensors.

**Figure 12:**
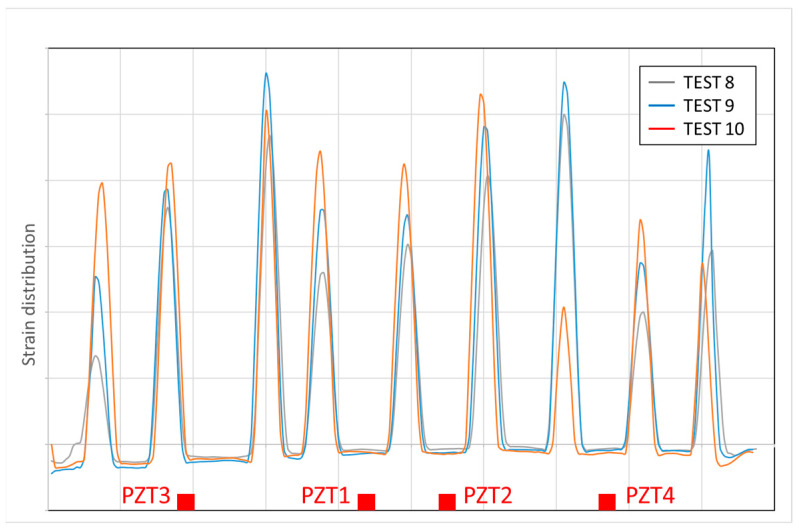
Concrete strain distribution.

Then, except for the surface crack which appears in Test 6 close to Sensor 6, the crack distribution is very similar for Tests 4 to 10, although the strain magnitudes progressively increase. However, for PZT Sensors 3 to 9, a considerable impedance variation is captured in Test 9. This is especially remarkable for Sensor 4. The simple observation of strain localization for Test 9 does not show any particular anomaly with respect to previous tests, but any internal phenomenon might be responsible for this variation obtained via hierarchical clustering. To obtain a deeper insight into it, the evolution of the strain profile during the loading along the length of the region of interest and to a depth corresponding to the location of the internal sensors is shown in Figure 12. The strain peaks clearly show the cracked areas and how they grew whenever the load level increased. It is interesting to note how the strain peak in the vicinity of Sensor 4 experiences a sudden drop from Test 9 to Test 10, which agrees with the previous comment about the anomaly identified with the clustering approach for Test 9. Similar to Specimen 1, the capacity of the EMI method to detect internal anomalies allows their identification before they extend to the surface. If Figure 11h is observed in detail, in the crack located on the left of Sensor 4, this drop is detected just in the region closer to the lower face. Although the beam was not loaded until failure, this extra anomaly might be the initiation of the debonding between the epoxy and concrete and a symptom of a near failure if the concrete had not been crushed before.

As for Specimen 1, the integration of the EMI and DIC approaches for Specimen 2 provided a robust and successful practical method for the SHM of FRP-strengthened RC beams, improving the conclusions of the independent analysis of each particular methodology.

## 5. Conclusions

Both DIC analysis and EMI technology, as fast-emerging technologies, have been applied successfully in different SHM applications. Whereas DIC allows the monitoring of strain profiles by correlating images captured with a digital camera, EMI has shown its damage detection capacity under different conditions.

The results of this work show the capabilities of combined EMI and DIC to discriminate the anomalies appearing in FRP-strengthened RC beams as evidence or early warnings of a possible future sudden brittle failure, which might have catastrophic consequences. Experimental studies on EBR FRP- and NSM FRP-strengthened specimens were carried out to understand their performance until failure using EMI and DIC observations.

An unsupervised hierarchical clustering technique was used to classify the impedance signals. Additionally, strain maps for the different damage stages of the analyzed specimens were evaluated using DIC. This study jointly using EMI and DIC results allowed us to more suitably analyze the responses of PZT sensors, leading to a better understanding of the failure mechanisms in these kinds of structures. The contributions of this study will be very useful regarding future applications of SHM or even for the implementation of damage numerical models for structures as complex as these.

## Figures and Tables

**Table 1:** Sequence of static loading tests for Specimen 1.

Test Number	Load Level	Test Code
1	0 kN	1
2	6 kN	2
3	14 kN	3
4	16 kN	
5	25.6 kN	
6	26.3 kN	4
7	31.3 kN	5
8	32 kN	6
9	38 kN	7
10	44 kN	8
11	47.9 kN	
12	53.4 kN	9
13	63.8 kN	
14	70 kN	
15	85.5 kN	
16	92 kN	10
17	94 kN	11

**Table 2:** Sequence of static loading tests for Specimen 2.

Test Number	Load Level
1	0 kN
2	8.6 kN
3	25 kN
4	36.5 kN
5	40 kN
6	43 kN
7	52.5 kN
8	60 kN
9	70 kN
10	80 kN
11	100 kN

## Data Availability

The data presented in this study are available upon request from the corresponding author. The data are not publicly available due to privacy reasons.
